# Effects of 3% Boric Acid Solution on Cutaneous *Candida albicans* Infection and Microecological Flora Mice

**DOI:** 10.3389/fmicb.2021.709880

**Published:** 2021-09-07

**Authors:** Qing Liu, Zhao Liu, Changlin Zhang, Yanyan Xu, Xiaojing Li, Hongqi Gao

**Affiliations:** ^1^Department of Clinical Medicine, Hebei University of Engineering, Handan, China; ^2^Department of Dermatology, Affiliated Hospital of Hebei University of Engineering, Handan, China

**Keywords:** boric acid, *Candida albicans*, mice, ITS, 16S rRNA, skin microecology

## Abstract

To determine the effect of 3% boric acid solution on cutaneous infections with *Candida albicans* (CA) in mice and its effect on skin microflora. Female mice were divided into three groups, with 18 mice in each group. Two injection sites were randomly selected, and 0.1 mL of CA mycelium suspension was injected into the epidermis and dermis of the back of mice. Group N was treated with sterile water for injection (SWFI). We observed the clinical manifestations, fungal fluorescence microscopic examination and colony count. Group B were hydropathically compressed with 3% boric acid solution for 30 min every 12 h. Group M was treated with SWFI, and group N was not treated. One week later, each group was observed with naked eyes, and skin samples were collected. The effect of boric acid on skin microflora was measured using Internal Transcribed Spacer Identification (ITS) and 16S rRNA genes. There were no significant changes in group M. In group B, the degree of skin injury was alleviated, the wounds healed markedly, and the exudate amount decreased. The effective rate of group B (83%) was significantly higher than that of group M (25%) (*P* < 0.05). The relative average abundance of *Candida* (*P* < 0.0001) and CA (*P* < 0.05) in group B was significantly lower than that in group M. Compared with group M, the microbial richness of group B changed little, but the diversity decreased. The flora structure of group B was significantly different from that of group M, but like that of group N. In group B, the abundance of *Proteobacteria* (*P* < 0.001), *Enterobacteriaceae* (*P* < 0.001), and *Escherichia-Shigella* (*P* < 0.001) was significantly greater, and the abundance of *Firmicutes* (*P* < 0.001), *Staphylococcaceae* (*P* < 0.001), and *Staphylococcus* (*P* < 0.001) were significantly lower. The 3% boric acid solution significantly reduced the symptoms of skin infection with *Candida albicans*. It inhibited the growth of *Candida albicans* and CA, reduced the diversity of skin microorganisms, increased the abundance of *Proteobacteria, Enterobacteriaceae, Escherichia-Shigella*, and reduced the abundance of *Firmicutes, Staphylococcaceae, Staphylococcus*.

## Introduction

There are more than 200 species of *Candida*, dozens of which have been proved to cause human candidiasis. Cutaneous candidiasis is the most common and most pathogenic (Sardi et al., 2013; Dardas et al., 2014).*Candida albicans* (CA) typically colonizes the surface of normal human skin and mucous membranes. The carriage rate of CA in normal people is as high as 14–45% (Perlorth et al., 2007). When immunological resistance is low or flora imbalances, CA often invades skin folds and causes cutaneous candidiasis, with a high incidence in summer and autumn. It is more common in infants, diabetics, obese, hyperhidrotic individuals, and those working in humid environments (Wang et al., 2012). In severe cases, *Candida* can cause deep infections. For immunosuppressed individuals, systemic infection is often life-threatening and difficult to treat, with mortality rates between 46 and 75% (Brown et al., 2012).

The treatment of superficial skin CA infection is mainly external use of drugs, once or twice a day. The course of treatment is 2–4 weeks. Imidazoles and acrylamines are preferred, and imidazoles include miconazole, bifonazole, ketoconazole and so on. Allylamines include terbinafine, butenafine, and naftifine. Others include morpholine, lira naphthyl ester (thiocarbamate), cyclopamine (cyclopyrone), imidazoles, and acrylamines (Katoh, 2009; Riachi, 2013). Patients with severe infections should be treated with combinations of oral medications. The partial effect of local treatment alone is unsatisfactory. The tissue selectivity of drugs is low. There are relatively many toxic side effects and adverse reactions, and drug resistance is possible. Therefore, there is an urgent need for alternative therapies.

As a common topical drug in dermatology, boric acid is less expensive, widely available, easy to use, and causes little irritation. *In vitro* tests showed that boric acid and boron-containing compounds inhibited *Candida*, *Trichophyton*, gram-positive bacteria, and gram-negative bacteria (Gerdon and Flynm, 2014). Its antibacterial activity is time-dependent and concentration-dependent (Iavazzo et al., 2011). A low concentration of boric acid inhibits pathogenic bacteria, while high concentrations of boric acid kill pathogenic bacteria (Brittingham and Wilson, 2014). Boric acid at 10–20 mg/mL inhibits almost all common bacteria or fungi (Hui et al., 2016). Boric acid has been used to treat cutaneous CA infections for more than one hundred years (Javad et al., 2015; Gao and Pan, 2016); nevertheless, its specific mechanisms are not clear but may involve the inhibition of mitochondrial enzyme activity and energy metabolism (Schmidt et al., 2018). Studies showed that boric acid increases the permeability of the pathogen cell wall, destroys cell membranes, and inhibits cell membrane formation (Brittingham and Wilson, 2014; Marrazzo et al., 2019). Nevertheless, the effect of boric acid on microflora has been rarely reported.

Therefore, in the present study, a mouse cutaneous CA infection model was established. ITS and 16S rRNA high-throughput sequencing were used to measure the improvement of clinical symptoms and skin microflora before and after treatment.

### Laboratory Animals and Strains

Healthy female ICR mice aged 6–8 weeks, weighing 22–24 g, were purchased from Beijing Weitong Lihua Experimental Animal Technology Co., Ltd. *Candida albicans* standard strain SC 5314 was purchased from the American Type Culture Collection.

### Reagents and Instruments

The 3% boric acid solution was provided by the Affiliated Hospital of Hebei Engineering University. The DNA extraction kit was purchased from MP Biomedicals (US). The library-building kit was provided by Bioo Scientific Corp. (US).

### Preparation of *Candida albicans* Liquid

The standard strain of CA (SC5314) stored at –4°C was thawed at room temperature. The concentration of bacteria was adjusted to 1.5 × 10^9^ colony-forming units CFU/mL through purification and activation of bacteria.

### Model Construction and Group Intervention

Mice were randomly divided into N (*n* = 18), M (*n* = 18), and B groups (*n* = 18). Chlorpromazine solution 0.2 mL was injected intramuscularly for anesthesia, hair on the back was shaved, and two injection sites were randomly selected. The mycelium suspension 1.5 × 10^9^ cells/0.1 mL was injected into the M and B groups, and the control group was treated with SWIF. Six mice were randomly selected to observe the clinical manifestations. Fungal fluorescence microscopy and colony count were used to determine whether the mouse skin CA infection model was successfully constructed.

On the fifth day after inoculation, 12 mice in each group were anesthetized by the above methods. Each mouse in group B was hydropathic compressed with six layers of sterile gauze and 3% boric acid solution for 30 min, once every 12 h. Group M was treated with SWIF, while the blank group was fed normally without treatment.

### Sample Collection

On the 7th day of treatment, an aseptic flocking cotton swab infiltrated with saline was repeatedly wiped at each mouse’s back in each group for 30 s to remove the microorganisms on the skin surface. We cut the cotton swab head into 2.0 mL aseptic frozen tubes, quick-frozen in liquid nitrogen, and stored at –80°C.

### Fluorescence Microscopic Examination of Fungi

Five days after inoculation, two mice were randomly selected from each group, a total of six mice. An aseptic cotton swab infiltrated with saline was used to wipe and rotate each mouse’s back in each group, spread on glass slides, and observed under a light microscope after adding potassium hydroxide solution dropwise.

### Colony Counts

Five days after inoculation, two mice were randomly selected from each group, a total of six mice. The infected tissue was quickly cut along the edge of the skin lesion with surgical scissors in the aseptic operating table, and the infected tissue was broken, centrifuged, diluted, and colony counted.

### Judgment of Curative Effect

The curative effect was judged by the standard as described (Jie et al., 2019). A markedly effective refers to the original skin lesion healing after 7 days of treatment; effective refers to the decrease of the original purulent exudate and improvement of skin lesion healing after 7 days of treatment. Ineffective means that the clinical symptoms are not significantly improved or even aggravated.

### Detection of Microflora Diversity

Skin samples frozen at –80°C were removed and treated with FastDNA SpinKitforSoil^®^ to extract the total DNA of skin microorganisms. The library was prepared by DNA purity and concentration detection, and PCR amplification. The corresponding regions of bacterial 16srRNA and fungal ITS primers for high-throughput sequencing PCR amplification were shown in Table 1. Finally, the Miseq PE300/NovaSeqPE250 platform of Illumina Company was used for sequencing, reads-splicing and filtering, OTUs clustering, bioinformatics analysis, and data processing.

**TABLE 1:** PCR Sequencing regions and primer sequences.

Sequencing area	Up-primer	Lower-stream primers
Bacteria 16S rDNA 338F_806R District	338F: ACTCCTACG GGAGGCAGCAG	806R: GGACTACH VGGGTWTCTAAT
Fungi ITS1F_ITS1F District	ITS1F: CTTGGTCATTT AGAGGAAGTAA	ITS1F: GCTGCGTTCT TCATCGATGC

### Statistical Analysis

SPSS23.0 software was used for statistical analysis. The measurement data were expressed as mean ± standard deviation (χ ± s). Analysis of variance or the Kruskal–Wallis test was used for comparisons among groups. The least-squares difference test was used to analyze the differences between the two groups further. The independent sample *t*-test was used for comparisons between the two groups. Differences were statistically significant when *P* < 0.05. We use mothur software to calculate alpha diversity indexes (Chao1, ACE, Shannon, and Simpson) and R language tools to create drawings.

### Clinical Manifestations

**FIGURE 1:**
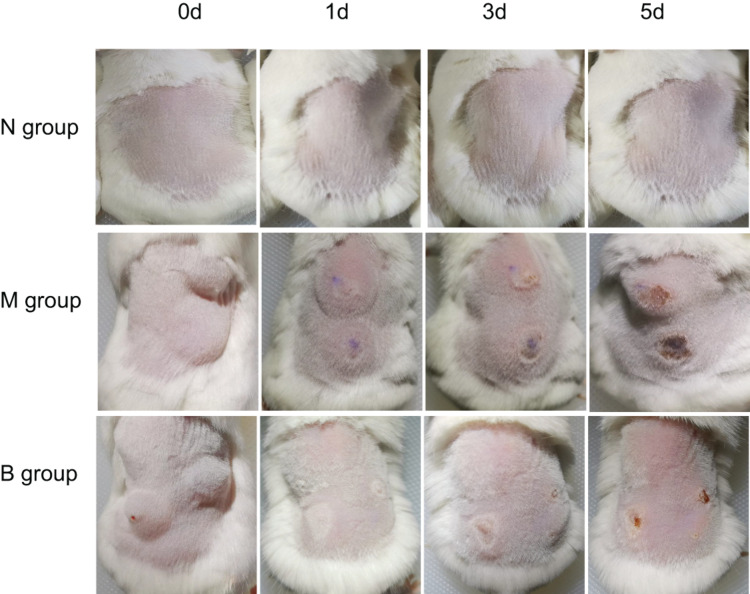
Skin changes from days 0 to 5 in each group.

After inoculating CA SC5314 (Figure 1), subcutaneous masses formed in M and B groups. Over time, the subcutaneous masses were gradually absorbed, white filmS appeared on the first day, and mild necrosis appeared on the third day, which was most apparent on the 5th day. Erosions and ulcers could be seen in the model. There were no changes in group N.

### Fluorescence Microscopic Examination of Fungi

**FIGURE 2:**
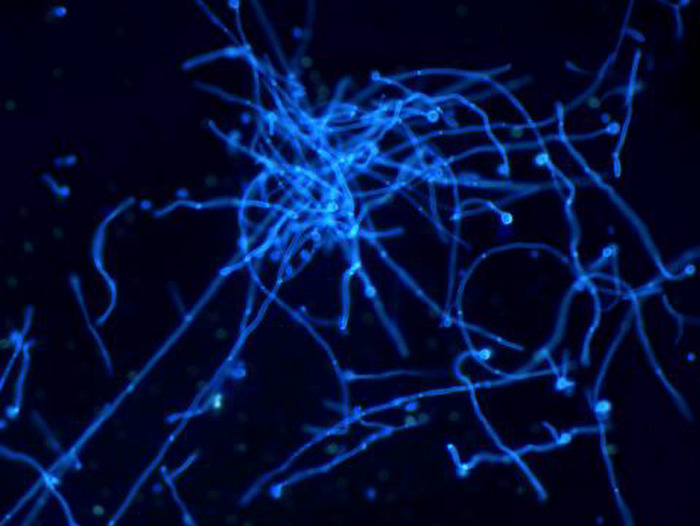
Direct skin microscopic results (×400 magnification).

On the 5th day after inoculation of CA SC5314 (Figure 2), evident agglomerated hyphae were found in both M and B groups. The microscopic examination of fungi in group N was negative.

### Colony Counts

**FIGURE 3:**
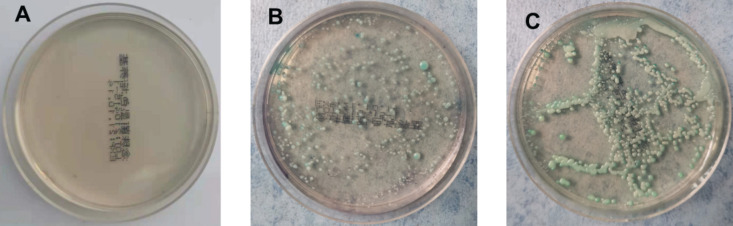
Color culture of *Candida albicans.* **(A)** Refers to group N, **(B)** refers to group M, and **(C)** refers to group B.

**FIGURE 4:**
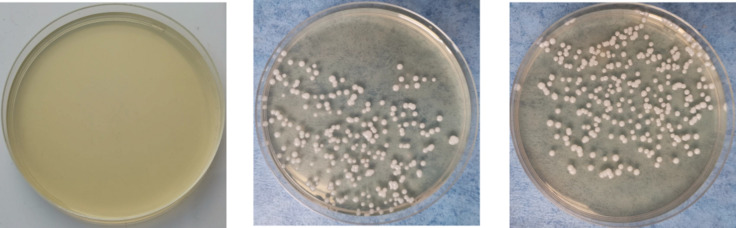
Growth of *Candida albicans* coated plate. **(A)** Refers to group N, **(B)** refers to group M, and **(C)** refers to group B.

On the 5th day after inoculation of CA SC5314 (Figure 3), CA was cultured in a chromogenic screening medium. CA appeared emerald-green with smooth colonies in M and B groups, while CA was negative in group N. Microscopic observation and counting showed that both M and B groups had higher fungal loads, about 10^5^–10^6^ CFU/g (Figure 4). We observed that the cutaneous infection model was successfully established from the three indicators of clinical manifestations, microscopic examination of fungi, and colony counts.

### Therapeutic Effect

**FIGURE 5:**
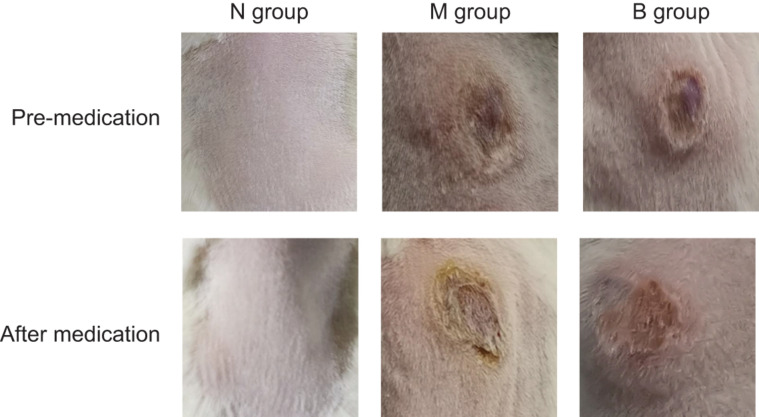
Skin conditions before and after treatment.

There was no irritation after treatment, and the skin lesions at the modeling site were observed with naked eyes on the 7th day (Figure 5). There was no apparent change in group N; however, erosions could be seen in group M, which was not significantly better than before treatment. The degree of injury was reduced in group B, suggesting that the wound underwent apparent healing without exudate. Treatment results are shown in Table 2. The number of markedly effective cases was 0; however, after 3% boric acid solution treatment, ten cases were effective, two cases were ineffective, and the effective rate was 83%. After SWFI, three cases were effective, nine were ineffective, and the effective rate was 25%. By comparison, the effective rate of group B was significantly higher than that of group M (*P* < 0.05), suggesting that 3% boric acid solution had a therapeutic effect on cutaneous CA infections.

**TABLE 2:** Comparison of therapeutic effects among groups.

Group	Markedly effective	Effective	Ineffective	Total	*P*
M	0	3	9	12	0.012
B	0	10	2	12	

### The Average Relative Abundance

A total of 72 skin samples were collected from 24 skin samples in each group, of which 44 skin samples were qualified and were sequenced using ITS. The bands of PCR amplification products of all skin samples in the blank group were too weak or were not detected, and no follow-up experiments were carried out. Considering that this phenomenon may have been related to fungi’s low content in mouse skin, it was challenging to obtain sufficient samples. Referring to the Unite database of ITS, the dominant fungus of genus level was *Candida* (95.40%), and the dominant fungus on the species level was CA (95.26%), consistent with the results of our fungal microscopic examination, further demonstrating that the mouse skin was infected with CA successfully. The average relative abundance of *Candida and* CA in each group was compared (Table 3). The *Candida* (*P* < 0.01) and CA (*P* < 0.05) of group B were significantly lower than those of group M, suggesting that boric acid had a therapeutic effect. The therapeutic effect was consistent with that of naked-eye observation.

**TABLE 3:** Comparison of abundance of *Candida* in each group.

Group	Candida	CA	*P*
M (*n* = 20)	99.83 ± 0.31	99.70 ± 0.33	0.00
B (*n* = 24)	91.15 ± 19.07	90.99 ± 19.05	0.04

### Alpha Diversity of Bacterial Flora

**FIGURE 6:**
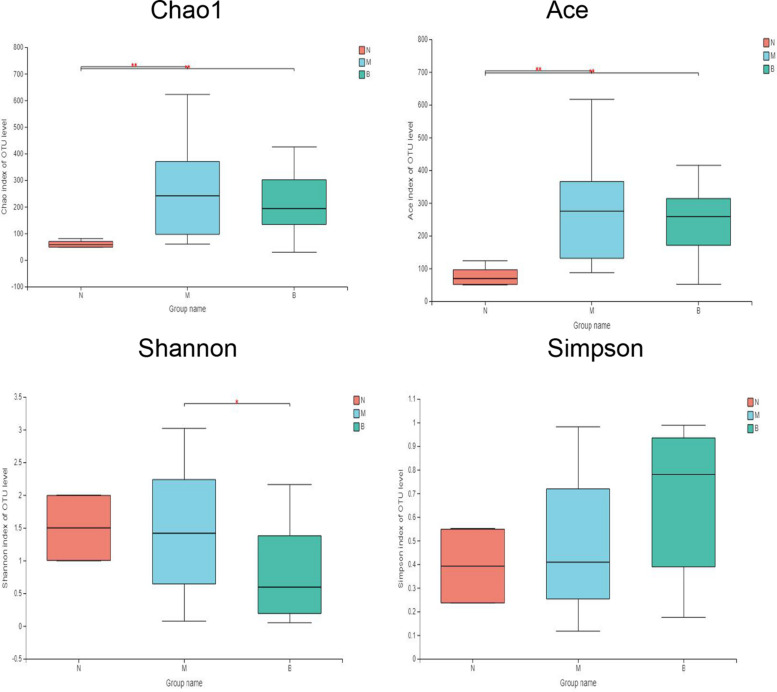
Comparative analysis of diversity indices of different treatment groups. *P ≤ 0.05; **P ≤ 0.01.

The diversity indices of the three groups are shown in Figure 6 (Li et al., 2013). Compared with group N, the Ace and Chao1 indexes of group M (*P* < 0.01) and group B (*P* < 0.01) were significantly increased, while there was no significant difference between group M and group B, suggesting that the flora richness of group M and group B was higher. This finding suggests that the microflora richness of the model and boric acid groups were higher. This may be related to skin infection with CA in both groups of mice. In comparing the diversity index, we found that the Shannon index of group B was significantly lower than that of group M (*P* < 0.05), and the Shannon index of group B was lower than that of group N (*P* = 0.126); however, the difference was not significant. The Simpson index in group B was higher than those of groups N (*P* = 0.094) and M (*P* = 0.054); however, the differences were not significant. These findings suggest that the skin microflora diversity of group B was decreased than that of the other two groups, and boric acid may reduce the bacterial flora diversity on the skin surface.

### Analysis at the Phylum Level

**FIGURE 7:**
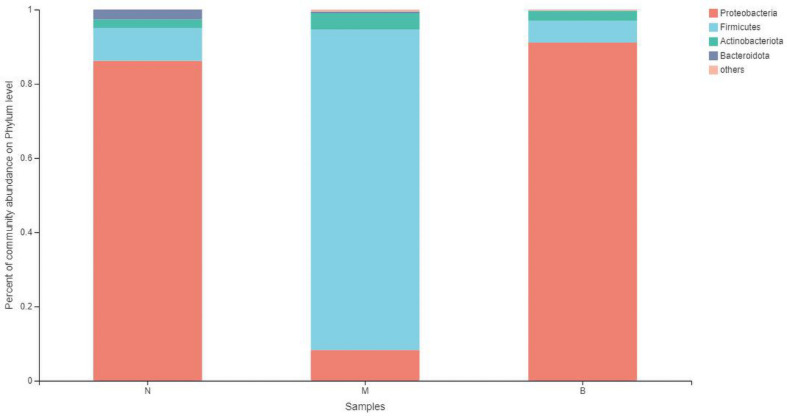
The abundance of phylum level.

At the phylum level (Figure 7), the community bar diagram showed that *Proteobacteria*, *Firmicutes*, *Actinobacteria*, *Bacteroidota* were dominant colonies in mouse skin. However, the abundance of these four bacteria in the three groups were significantly different, 86.19, 8.87, 2.31 and 2.63% in group N, 8.26, 86.40, 4.34 and 0.46% in group M, and 91.07, 5.86, 2.67, and 0.09% in group B, respectively.

**FIGURE 8:**
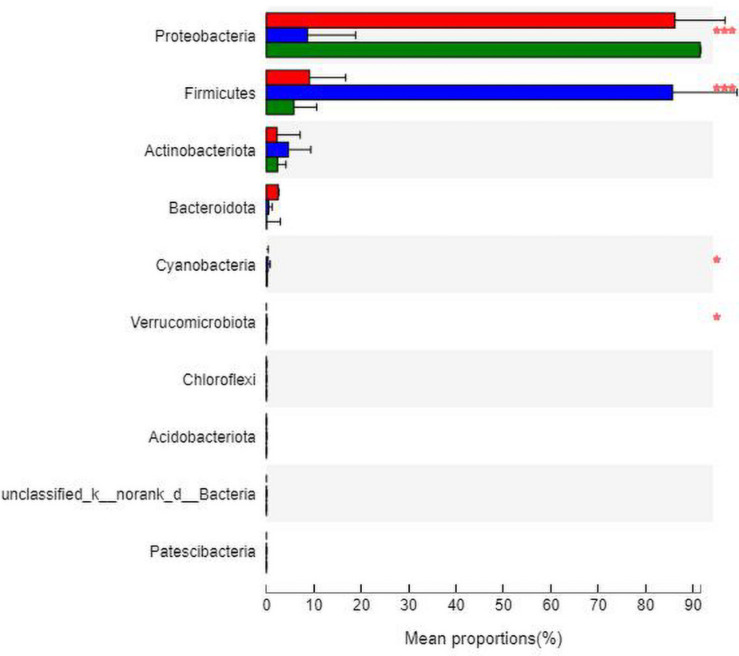
Analysis of the differences at the phylum level. *P ≤ 0.05; ***P ≤ 0.001.

The analysis of species differences is shown in Figure 8. Compared with group N, the *Cyanobacteria* and *Verrucomicrobiota* in groups M (*P* < 0.05) and B (*P* < 0.05) were significantly higher. Compared with group M, the *Proteobacteria* in groups N (*P* < 0.001) and B (*P* < 0.001) were significantly higher. At the same time, the *Firmicutes* were significantly lower in groups N (*P* < 0.001) and B (*P* < 0.001).

### Analysis at the Family Level

**FIGURE 9:**
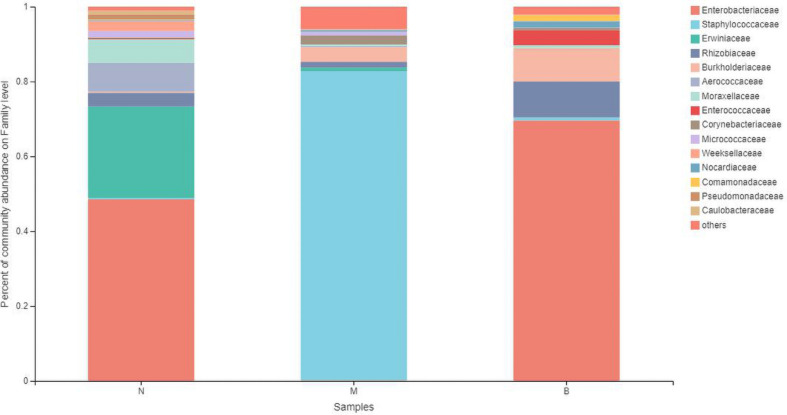
The abundance of the family level.

At the family level (Figure 9), *Enterobacteriaceae* was the most abundant species in groups N and B, with proportions of 48.52 and 69.58%, respectively. *Enterobacteriaceae* only accounted for 0.14% in group M. *Staphylococcaceae* was the most abundant species in group M, with a proportion of 82.61%, and *Staphylococcaceae* abundances were 0.35 and 0.77% in groups N and B, respectively.

**FIGURE 10:**
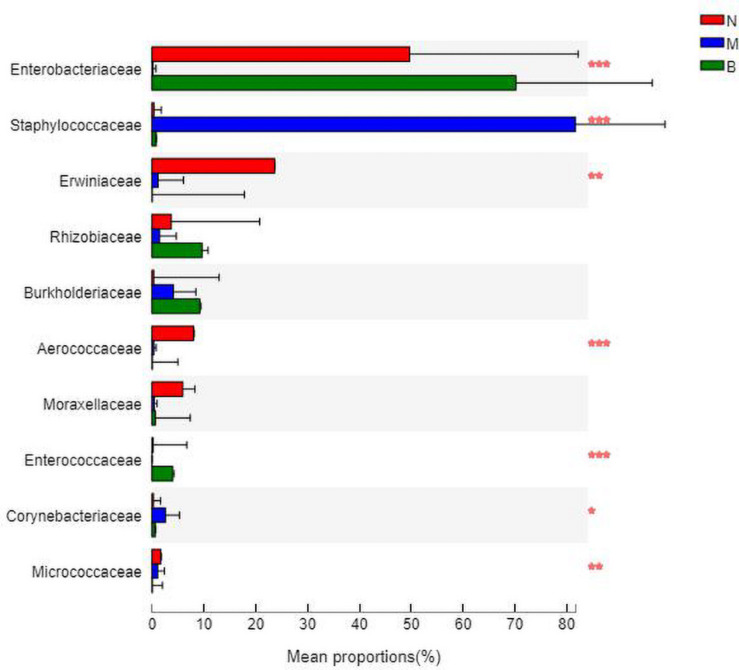
Analysis of the differences at the level of the family level. *P ≤ 0.05; **P ≤ 0.01; ***P ≤ 0.001.

The analysis of species differences is shown in Figure 10. *Erwiniaceae* and *Aerococcaceae* in group N were significantly increased (*P* < 0.01). In group M, *Enterobacteriaceae* was significantly decreased, while *Staphylococcaceae* and *Corynebacteriaceae* were significantly increased (*P* < 0.01). In group B, Enterococcaceae was significantly increased, and Micrococcaceae was significantly decreased (*P* < 0.01).

### Analysis at the Genus Level

**FIGURE 11:**
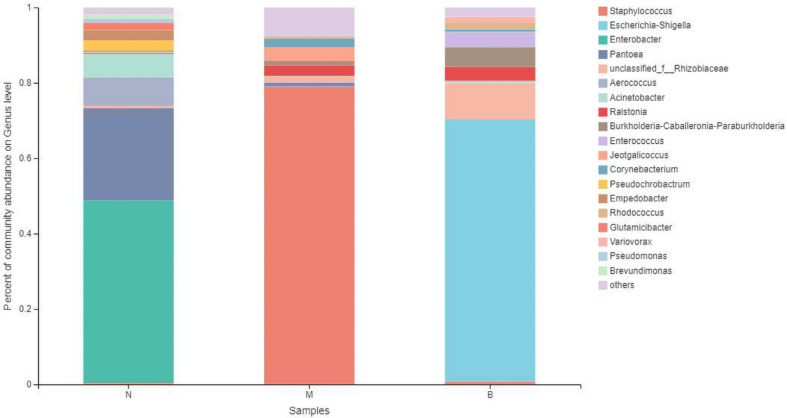
The abundance of the genus level.

At the genus level (Figure 11), *Enterobacter* was the most abundant species in group N, accounting for 48.49%, while only 0.12 and 0% in groups M and B, respectively. The dominant species in group M was *Staphylococcus*, accounting for 79.05% and only 0.34 and 0.70% in groups N and B, respectively. The most abundant strain in group B was *Escherichia*, followed by *Shigella*, with a proportion of 69.58%, while in groups B and M, both were close to 0%.

**FIGURE 12:**
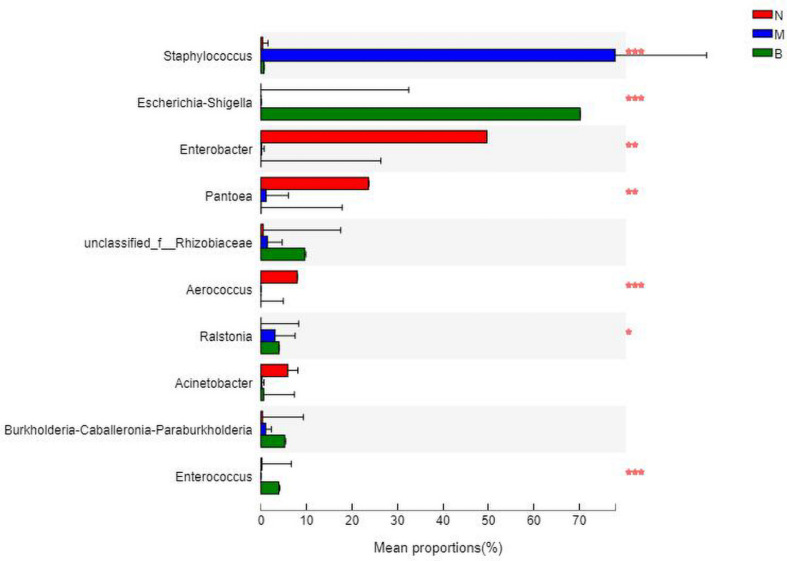
Analysis of the differences at the level of the genus level. *P ≤ 0.05; **P ≤ 0.01; ***P ≤ 0.001.

The analysis of species differences is shown in Figure 12. In group B, *Enterobacter*, *Pantoea*, and *Aerococcus* were significantly increased (*P* < 0.01), and *Ralstonia* was significantly decreased (*P* < 0.05). *Staphylococcus* in group M was significantly increased (*P* < 0.01). *Escherichia-Shigella* and *Enterobacter* in group B were significantly increased (*P* < 0.01).

### Community Heatmap Map

**FIGURE 13:**
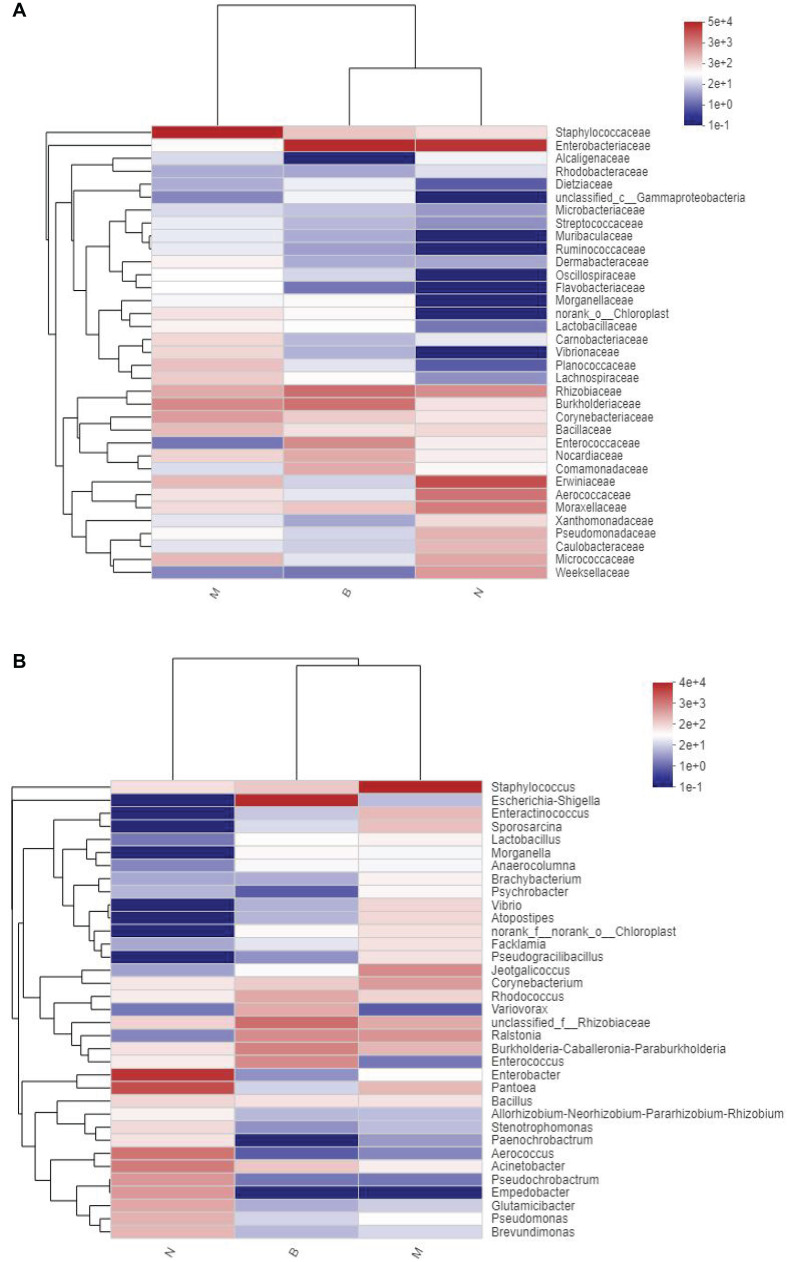
Distribution Heatmap of microbial communities in each group. **(A)** Refers to the family level; **(B)** refers to the genus level.

At the family and genus level, a heatmap of species clustering was drawn (Figure 13). We found that the dominant bacteria genera of the microorganisms in the three groups were different. Most of groups N and B were from *Proteobacteria*, and most of group M were from *Firmicutes*. At the family level, the cross-clustering between group N and group B indicated that the dominant bacteria family composition of the two groups was more similar but different from that of group M. However, at the genus level, the cross-clustering between groups M and B suggested that the dominant bacteria genus composition of the two groups was more similar. However, the dominant bacterial genus of group B showed a changing trend of transformation to group N, suggesting that boric acid may improve the structure of the skin bacteria community and develop toward a healthy and beneficial trend.

## Discussion

With the widespread use of antibiotics, the prevalence of diabetes and obesity, dentures, and other factors, the risk of cutaneous CA infection has become significant. These infections include chronic skin candidiasis, *Candida* intertriginous rash, angular stomatitis, oral candidiasis, candidal onychomycosis and onychomycosis, vaginitis, and balanitis, and others (Spampinato and Leonardi, 2013).

Animal models play essential roles in the study of CA pathogenicity, immunity, drug screening, and new drug research and development. In recent years, there have been several established models, including a systemic *Candida* infection model (Fouts et al., 2012), an oral candidiasis infection model (Solis and Filler, 2011), a vaginal candidiasis infection model (Liu and Wu, 2019), a *Candida* keratitis infection model (Iliev et al., 2012), a lower respiratory tract infection model (Xie, 2011), and a non-mammalian infection model (Sampaio et al., 2018). Nevertheless, there are only a few reports of a cutaneous Candida infection model, including two specific methods. The first method is the classical method of directly applying CA solution to the exfoliated epidermis, described by Gaspari (Santus et al., 2018). In this study, we selected the second method of direct intradermal injection discovered by Fang (Fang et al., 2020) to induce the formation of a CA infection model. Five days after inoculation, white membranes, necrosis, erosions, and ulcers appeared on the back of mice. Fungal hyphae could be seen under a fluorescence microscope, smooth emerald green colonies were shown on the CA screening medium, and the colony culture count reached 10^5^–10^6^ CFU/g.

Boron exists in the form of boric acid at physiological pH (Larsen et al., 2018). Boric acid, a common antibacterial agent (Schmidt et al., 2010), is widely used to treat fungal infections. In recent years, boric acid has been found to have therapeutic effects on cutaneous CA infections, and relevant research has been incorporated into clinical guidelines (Pointer and Schmidt, 2016). Compared with traditional antifungal drugs, adverse reactions associated with boric acid are rare (Powell et al., 2019), there are no interactions with other drugs, and it does not cause microbial drug resistance. Boric acid has antibacterial activity against various yeasts, limiting the growth of CA and inhibiting smooth *Candida albicans* (Pointer et al., 2015).

Nevertheless, its mechanisms of action are not clear. They may involve destroying the cytoskeleton and preventing actin recombination, resulting in abnormal mycelium development, thereby inhibiting mycelium growth (Larsen et al., 2018). Some investigators found that boric acid inhibits glyceraldehyde 3-phosphate dehydrogenase (an NAD glycolysis-dependent enzyme) and ethanol dehydrogenase (an NADH-dependent fermentation enzyme) in CA, thereby inhibiting NAD/NADH-dependent reactions in carbohydrate metabolism, affecting energy metabolism, promoting ethanol production, and increasing the sensitivity of CA to ethanol toxicity (Pointer and Schmidt, 2016). In the present study, we found that, after boric acid treatment, the skin showed no irritation, and the degree of injury was alleviated, suggesting wound healing. ITS sequencing analysis showed that the abundance of *Candida* and CA in group B was significantly lower, further suggesting that boric acid inhibited *Candida* and CA growth. The effects of boric acid on the microbial richness, diversity, and composition of microflora in the skin of mice infected with CA were analyzed using the 16S rRNA sequencing technique. We found that boric acid improves the skin microecology and reduced skin microbial diversity but had little effect on richness. Boric acid increased the abundance of *Proteobacteria*, *Enterobacteriaceae*, and *Escherichia-Shigella and* reduced *Firmicutes*, *Staphylococcaceae*, and *Staphylococcus*.

There are large and complex microbial populations on the skin’s surface, up to more than 10,000 (Arnold et al., 2019), all of which are important for maintaining the balance of skin microecology. There is competition or restriction in the normalskin micro-ecosystem. The abnormal increase, decrease, or even disappearance of skin microflora leads to the destruction of skin microecology, called skin microecological imbalance. The microecological imbalance may directly lead to infection reaction, divided into endogenous and exogenous types. The endogenous type comes from proportion imbalances, localized metastases, and double infections with normal microbiota. The exogenous type comes from the invading competition of foreign bacteria, which greatly reduces or even eradicates resident bacteria, resulting in skin damage (Wu et al., 2016; Eisenstein, 2020). The microbial population of adult skin is highly personalized, and the composition of strains is stable. Living with trillions of microbes is not without risk (Oh et al., 2014; Kuhbacher et al., 2017). CA is usually present in human skin; however, it is not a symbiotic bacteria on mouse skin surfaces (Iliev et al., 2012). ITS sequencing showed that boric acid decreased the abundance of *Candida*; however, it did not reach the strain level; therefore, further metagenomic sequencing was needed. *Escherichia-Shigella* belongs to Proteobacteria, Enterobacteriaceae. *Staphylococcus* belongs to Staphylococcaceae, Firmicutes. The 16S rRNA sequencing showed a significant difference in the flora structure between the boric acid and the model groups; however, the former was like the blank group. Boric acid may play a protective role by adjusting the microecological skin flora to achieve dynamic balance.

In summary, by constructing a cutaneous CA infection model in mice and measuring the therapeutic effect of 3% boric acid on skin microflora, we concluded that boric acid inhibited Candida and CA’s growth, reduced microbial diversity, and improved the microecological flora in mouse skin. This study will expand the understanding of boric acid as an antifungal and provide a basis for developing new antifungal drugs.

## Data Availability Statement

The data presented in the study are deposited in the Sequence Read Archive (SRA) repository, accession number SUB9815704.

## Ethics Statement

The animal study was reviewed and approved by the Biomedical Ethics Committee of Medical School of Hebei University of Engineering.

## Author Contributions

QL wrote the manuscript. XL and HG revised the manuscript. ZL, CZ, and YX gave some helpful suggestions. All authors contributed to manuscript revision, read, and approved the submitted version.

## Conflict of Interest

The authors declare that the research was conducted in the absence of any commercial or financial relationships that could be construed as a potential conflict of interest.

## Publisher’s Note

All claims expressed in this article are solely those of the authors and do not necessarily represent those of their affiliated organizations, or those of the publisher, the editors and the reviewers. Any product that may be evaluated in this article, or claim that may be made by its manufacturer, is not guaranteed or endorsed by the publisher.
